# Radiomic and Clinical–Pathological Factors Predictive of Postoperative Recurrence in Lung Neuroendocrine Tumors: A Pilot Study

**DOI:** 10.3390/cancers17233812

**Published:** 2025-11-28

**Authors:** Piero Paravani, Michela Polici, Giulia Arrivi, Alessandra Siciliani, Massimiliano Mancini, Rossella Mazzilli, Virginia Zamponi, Maurizio Martiradonna, Federica Palmeri, Beatrice Trabalza Marinucci, Francesco Panzuto, Matteo Tiracorrendo, Antonio D’Andrilli, Mohsen Ibrahim, Damiano Caruso, Antongiulio Faggiano

**Affiliations:** 1 Endocrinology Unit, Department of Clinical and Molecular Medicine, Sapienza University of Rome, Sant’Andrea Hospital, ENETS Center of Excellence, 00189 Rome, Italy; piero.paravani@uniroma1.it (P.P.); rossella.mazzilli@uniroma1.it (R.M.); virginia.zamponi@uniroma1.it (V.Z.); maurizio.martiradonna@uniroma1.it (M.M.); 2 Radiology Unit, Department of Medical Surgical Sciences and Translational Medicine, Sapienza University of Rome, Sant’Andrea Hospital, 00189 Rome, Italy; michela.polici@uniroma1.it (M.P.); federica.palmeri@uniroma1.it (F.P.); damiano.caruso@uniroma1.it (D.C.); 3 PhD School in Traslational Medicine and Oncology, Department of Medical and Surgical Sciences and Translational Medicine, Faculty of Medicine and Psychology, Sapienza University of Rome, 00185 Rome, Italy; 4 Oncology Unit, Department of Clinical and Molecular Medicine, Sapienza University of Rome, Sant’ Andrea Hospital, 00189 Rome, Italy; giulia.arrivi@uniroma1.it; 5 Division of Thoracic Surgery, Department of Medical and Surgical Sciences and Translational Medicine, Sapienza University of Rome, Sant’Andrea Hospital, 00189 Rome, Italy; asiciliani@ospedalesantandrea.it (A.S.); beatrice.trabalzamarinucci@uniroma1.it (B.T.M.); matteo.tiracorrendo@uniroma1.it (M.T.); antonio.dandrilli@uniroma1.it (A.D.); mohsen.ibrahim@uniroma1.it (M.I.); 6 Morphologic and Molecular Pathology Unit, Sant’Andrea Hospital, 00189 Rome, Italy; mamancini@ospedalesantandrea.it; 7 Department of Medical-Surgical Sciences and Translational Medicine, Sapienza University of Rome, Digestive Disease Unit, ENETS Center of Excellence, Sant’Andrea University Hospital, 00189 Rome, Italy; francesco.panzuto@uniroma1.it

**Keywords:** lung neuroendocrine tumors, lung NET, NET, carcinoids, radiomics, prognostic factors

## Abstract

Background/Objectives: Neuroendocrine tumors (NETs) of the lung account for about 30% of NETs. In localized and locally advanced forms, radical surgical resection is the standard of care. Although considered indolent tumors, they appear to be susceptible to post-surgical recurrence, with rates differing between typical and atypical carcinoid. Although still debated, several clinicopathologic factors are potentially associated with recurrence. The aim of this retrospective/prospective observational study is to evaluate the predictive role of clinicopathological factors and radiomics features in patients with NET of the lung. Methods: From January 2021 to April 2024, 45 consecutive patients who underwent radical (R0) surgery for lung NET at the ENETS Center of Excellence of the Sant’Andrea Hospital were enrolled, all with at least 12 months of postoperative follow-up and availability of preoperative unenhanced chest CT. Clinicopathologic and radiomic factors were considered (107 radiomic features). Of the individual characteristics, the impact on recurrence was assessed by univariate logistic regression. Results: Among the 45 patients included, 4 patients (8.9%) experienced disease recurrence. Among the clinicopathological features, major age at diagnosis (*p* = 0.020), atypical carcinoid (*p* = 0.010), presence of functional syndrome (*p* = 0.002), advanced stage at diagnosis (*p* = 0.013), necrosis (*p* = 0.017) higher Ki-67 (*p* = 0.001), higher mitotic count (*p* = 0.006), and pathologic lymph node (*p* = 0.006) were associated with disease recurrence. Three radiomic features were found to predict recurrence: DependenceEntropy (*p* = 0.049), DependenceNonUniformityNormalized (*p* = 0.024), and Elongation (*p* = 0.039). In this preliminary analysis, multivariate analysis was not performed due to the small sample size. Conclusions: This study has shown that radiomics can be a valuable tool in predicting recurrence. Currently, to our knowledge, no other studies on the possible application of radiomics as prognostic factors in patients with lung NET have been published. These encouraging findings warrant further investigations with larger, multicenter cohorts to validate these results and implement them by constructing a predictive model of recurrence.

## Simple Summary

Neuroendocrine tumors of the lung account for 30% of all neuroendocrine neoplasms. The first-line treatment, when feasible, is surgery. The behavior of these tumors is very heterogeneous, so many studies have focused on the search for prognostic clinical–pathological factors. Radiomics, due to its proven capabilities, is increasingly used in oncology. Several studies have shown that it can be a very valuable tool to help clinicians identify and predict response to treatment in the field of neuroendocrine neoplasms. In this retrospective/prospective single-center study, we evaluated the applicability of this method in the field of neuroendocrine lung neoplasms for the first time. Several clinical–pathological factors such as age, histotype, and advanced stage were confirmed as negative prognostic factors, and some radiomic features were found to be predictors of recurrence.

## 1. Introduction

Neuroendocrine tumors (NETs) of the lung are a heterogeneous group of neoplasms originating from the neuroendocrine cells within the bronchial epithelium. These tumors are generally slowly proliferating but with a variable degree of clinical aggressiveness and prognosis. Overall, lungs represent the first primary site of origin of NETs, harboring about 25% of the total [1]. Data from the Surveillance, Epidemiology, and End Results (SEERs) US registries show that, although rare, the incidence of these neoplasms has significantly increased in the last fifty years, from 0.35 cases per year per 100,000 people in 1973 to 1.62 cases in 2012 [2]. According to the last WHO classification of thoracic NENs, among the lung NET histotypes, we can distinguish typical carcinoids (TCs) and atypical carcinoids (ACs) on the basis of mitotic count and presence of necrosis. TCs are considered low-grade NETs and are the most common form, while ACs are intermediate-grade tumors displaying more aggressive biological behavior. The management of these neoplasms is complex and requires a multidisciplinary approach. The ratio of TCs to ACs is approximately 8:1 to 10:1 [3], with TCs being the most frequently observed form. The median age at diagnosis is around 45 years for TCs and approximately ten years older for ACs. While factors like smoking have been investigated, the most commonly recognized underlying genetic cause is the presence of the MEN1 gene mutation in some cases. For diagnostic purposes, imaging techniques such as contrast-enhanced Chest CT are the standard for morphological evaluation, while functional characterization relies increasingly on PET with ^68^Ga-DOTA peptides to assess Somatostatin Receptor expression, which guides both staging and potential therapeutic options like Somatostatin Analogs or Peptide Receptor Radionuclide Therapy (PRRT) [4]. However, the prediction of recurrence remains imprecise. Despite the acknowledged prognostic value of histopathological factors (mitotic count and Ki-67 index) and clinical staging, these markers sometimes fail to accurately capture the full biological aggressiveness of the tumor. This leads to cases where a histologically indolent TC exhibits aggressive progression, or, conversely, an AC shows an unexpectedly benign course [5]. This inherent uncertainty in predicting individual patient outcomes highlights the urgent need for non-invasive, quantifiable biomarkers that can characterize the entire tumor volume’s heterogeneity and proliferation potential in vivo. In the localized and locally advanced forms of lung NETs, the standard of care is surgery, with the goal of totally removing pathologic tissue and preserving adjacent healthy lung tissue [6,7,8]. Although they are considered indolent forms, they are susceptible to recurrence with a recurrence rate of about 7% in TCs and 35% in ACs, represented by a local relapse (primary site or lymph nodes) in one third of cases [9,10]. The role of adjuvant treatment is still debated, but it may be indicated, after a risk/benefit assessment, in selected patients, such as those with AC and major lymph node involvement (N2 lymph node-positive). Given the variable course of these tumors, several studies focused on the study of predictive factors of postoperative recurrence, suggesting a role for male sex [11], advanced age at diagnosis [12], atypical histotype [13], stage [5], and high Ki-67 index [14]. By combining these factors, different predictive models have been proposed with good performance [11] and await validation in a prospective setting. Several innovative methods are currently being studied that may provide new assessment tools to be added to the classic factors used. These approaches include both the development of more representative preclinical platforms and new therapeutic strategies. For example, patient-derived xenograft (PDX) models provide a robust preclinical platform that preserves the genetic and phenotypic heterogeneity of tumors, mirroring their pathological and genetic characteristics, and are therefore valuable for studying progression and developing new therapies [15]. At the same time, a key element of the lung tumor microenvironment is Cancer-Associated Fibroblasts (CAFs), which contribute to tumor progression and metastasis. Despite their importance, the heterogeneity of CAFs is largely unknown, and the lack of specific markers makes them difficult to target therapeutically [16]. On the therapeutic side, while conventional therapies for metastatic lung cancer (TKI, chemotherapy, and radiotherapy) face limitations such as indiscriminate toxicity and drug resistance, nanotechnology is emerging as a critical solution. Nano-formulations and nanostructures are being developed to enable targeted drug delivery and enhanced radio-sensitization [17]. Among innovative assessment methods, radiomics represents an innovative methodology, allowing us to obtain quantitative descriptors from standard medical images, such as CT, MRI, or PET, to potentially improve the diagnostic-therapeutic approach of cancer. These features capture the lesion’s internal heterogeneity, shape, and texture, potentially correlating with the underlying tumor biology and aggressiveness in vivo. A clinico-radiomic model explicitly combines these quantitative imaging features with conventional clinical and pathological data to build a more robust and personalized prediction tool. These features could be used to capture disease properties or to monitor them throughout the duration of treatments. For instance, it has been demonstrated that the lesion’s biological heterogeneity influences its aggressiveness and that radiomics features are strongly correlated with lesion heterogeneity both within the lesion and at the cellular level. [18]. Radiomics features can be divided into the following categories: statistical, which include histogram-based and texture-based; model-based; transform-based; and shape-based [19]. Features can be extracted from 2-dimensional regions of interest (ROIs), which is reductive considering only the most representative slice, or 3-dimensional volumes of interest (VOIs), which is the most recommended way to segment the lesion, including the entire volume [20].

Recently, it has been demonstrated a possible role of some models based on radiomics features has been demonstrated in the differential diagnosis of pancreatic lesions [21], in predicting tumor grading [22], in response prediction to specific therapy [23,24], or even prognosis in patients operated for pancreatic NETs [25]. Regarding lung NETs, few data are available, and most studies focused on the differential diagnosis of pulmonary nodules of indeterminate nature. There is a significant gap in the literature concerning the predictive role of radiomics for postoperative recurrence in surgically resected lung NETs. The objective of the present study was to investigate various clinical–pathological factors and radiomic factors in order to identify potential prognostic factors.

## 2. Materials and Methods

### 2.1. Study Design and Study Population

This is a retrospective/prospective observational study involving adult patients (age > 18 years) with a histological diagnosis of pulmonary NETs (typical and atypical carcinoid), admitted to the ENETS Centre of Excellence at Sant’Andrea Hospital between January 2021 and April 2024.

The study is divided into two phases:

1. Retrospective Phase: This phase retrospectively included all patients who underwent radical surgery (R0) for localized or locally advanced pulmonary NETs between January 2021 and September 2023.
2. Prospective Phase: This phase prospectively included patients undergoing radical surgery (R0) with the same characteristics, operated on from October 2023 up to April 2024, and who had at least 12 months of postoperative follow-up.

Clinicopathological data were collected for all patients, including demographic information (sex, age at diagnosis, and smoking history) and histopathological characteristics (Ki-67 index, mitotic count, stage at diagnosis, intra-parenchymal tumor location, side of the primary tumor, and TTF-1 immunostaining). All patients had a preoperative unenhanced CT scan showing segmentable lung nodules, excluding all CT exams affected by motion artifacts.

All data were gathered during the first follow-up visit after surgery. Additionally, preoperative unenhanced chest CT images were used to extract and analyze radiomic features.

The follow-up protocol has not been modified from the standards used in our Center. In particular, there were no differences in follow-up between patients included in the retrospective phase and patients enrolled in the prospective phase. Patients were monitored through radiological examinations and follow-up visits alternating between total body CT and chest HRCT at 3, 6, and 12 months after surgery, and then every 6 months thereafter. Clinical visits were performed every six months throughout the duration of the study.

Disease recurrence was defined as the first objective evidence of malignant disease reappearance after radical (R0) surgical resection. This includes local recurrence (tumor regrowth at the primary surgical site or bronchial stump), regional recurrence (new or progressive tumor involvement in hilar or mediastinal lymph nodes), and distant metastasis (the appearance of lesions in organs remote from the chest, such as the liver or bones).

The aim of this study was to evaluate predictive factors for recurrence in patients with lung NET undergoing radical surgery using clinicopathological features and radiomic features.

### 2.2. CT Evaluation

All CT examinations were performed using 64-layer equipment (GE Revolution EVO 64 Slice CT Scanner, GE Medical Systems, Milwaukee, WI, USA). All patients were examined in the supine position, with cranio-caudal acquisitions.

The acquisition parameters were as follows: X-ray tube voltage 120 kVp; mAs between 130 and 260 with SMART mA modulation, from GE Healthcare; collimator 64 × 0.625 mm; pitch 0.984; scan time 0.6 s; image reconstruction thickness at 1.25 mm using both soft tissue and bone filters in the reconstructions.

### 2.3. Tumor Volumetric Segmentation and Radiomic Extraction Characteristics

**Figure 1:**
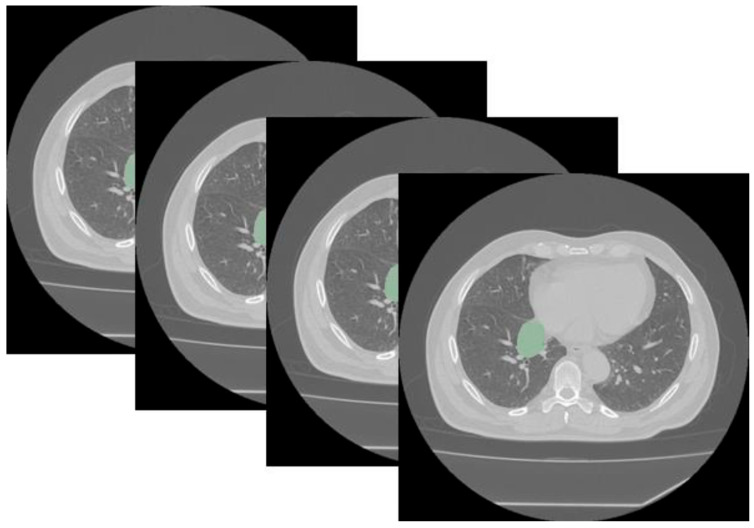
Segmentation using Slicer (version 5.6.2) of a typical central bronchial carcinoid visualized with a lung window (the segmented tumor is shown in green). Segmentation was performed in all cases using a mediastinal window.

Segmentation was performed manually by an experienced radiologist operator who drew a volumetric region of interest (VOI) of the main evaluable thoracic lesion by using the unenhanced phase of the preoperative basal CT scan. Consistency during manual segmentation was ensured by adhering to rigid departmental protocols and standard guidelines, which mandated the strict inclusion of only the lesion while avoiding adjacent structures like bronchi or blood vessels (Figure 1).

Open-source software, 3D Slicer (version 5.6.2, https://www.slicer.org, accessed on 4 June 2024) with the 3D Slicer Radiomics extension, was used for feature segmentation and extraction. In this study, an isotropic voxel size of 3 mm^3^ is used. Thus, 107 radiomics features were extracted using the soft tissue window of unenhanced chest CT, including first- and second-order features: 19 first-order, 13 2D and 3D shape, 16 features Gray-Level Size Zone Matrix (GLSZM), 5 features Neighboring Gray Tone Difference Matrix (NGTDM), 14 features Gray-Level Dependence Matrix (GLDM), 24 features Gray-Level Co-occurrence Matrix (GLCM), and 16 features Gray-Level Run Length Matrix (GLRLM).

### 2.4. Surgery

All patients underwent surgery by lateral muscle-sparing thoracotomy on the fifth intercostal space. This was a minimally invasive approach (6 to 8 cm incision) in most cases. Intraoperative one-lung ventilation, with a double-lumen endotracheal tube, was used. Patients underwent segmentectomy, lobectomy, bilobectomy, or sleeve resection according to tumor size and location. Systematic hilo-mediastinal lymphadenectomy was always performed. Surgical samples were sent for histological analysis.

In case of airway infiltration at the origin of the lobar bronchus, sleeve resection was performed, preparing and sectioning the main bronchus upstream and downstream of the infiltrated tract. Then, after en bloc removal of the lobe with the infiltrated portion of the main bronchus, an end-to-end anastomosis of the remaining main bronchus stump with the lobar bronchus of the safe remaining uninvolved lobe was performed with a running polydiaxone (PDS) 4-0 suture on the posterior-membranous wall and interrupted sutures on the anterior cartilaginous wall [26].

One patient received a Carinal Right Upper Sleeve Lobectomy because the tumor infiltrated the right main bronchus up to the tracheo-bronchial angle: the distal trachea was transected first above the area of infiltration, and the bronchus intermedius was then transected distally. The specimen, including the right upper lobe, carina, and right main bronchus, was removed. An end-to-end anastomosis between the tracheal stump and the left main bronchus was then performed. An end-to-side anastomosis between the bronchus intermedius and the medial aspect of the left main bronchus was then performed, thus allowing right middle and lower lobe preservation [27].

The anastomosis was then covered by a previously prepared vascularized intercostal muscle flap [28].

At the end, a chest tube was inserted through the VIII intercostal space and connected to a one-way drainage system.

All patients received chest X-rays on postoperative day 1 and blood test analyses.

All patients started pulmonary rehabilitation programs (mobilization and respiratory exercises) on postoperative day 1.

Patients who underwent bronchial resection-anastomosis (sleeve resections) received postoperative bronchoscopy before discharge and 1-3-6-12 months after surgery.

### 2.5. Statistical Analysis

Statistical analysis was performed by using SPSS version 27.0 (SPSS Inc. Chicago, IL, USA) and MedCalc Statistical Software version 17.9.7 (MedCalc Software bvba, Ostend, Belgium), with a *p*-value < 0.05 considered statistically significant.

All texture features were analyzed with receiver operating characteristic (ROC) curves, and the Area Under the Curve (AUC) was calculated to predict the performance of texture analysis. Recurrence was treated as a dichotomous outcome variable (Recurrence Yes/No). No Cox proportional hazards model or Kaplan–Meier analyses were performed on this data. Univariate logistic regression analysis was used to assess whether individual radiomics features and clinical pathological features were predictors of recurrence. Given the small sample size and the low number of events, which pose a high risk of overfitting, as the accepted rule of thumb is 10 events per variable (EPV), multivariate analysis was not performed in this preliminary analysis.

## 3. Results

### 3.1. Clinicopathologic Characteristics

**Figure 2:**
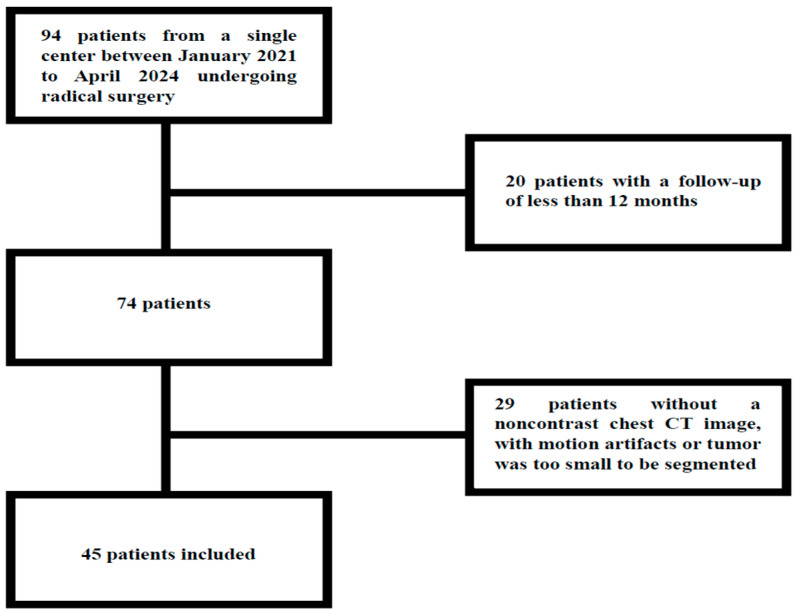
Study flow-chart.

From a total of 94 consecutive patients with lung NET who underwent radical surgery, 45 patients were enrolled in the study, of whom 32 (71.1%) were female and 13 (28.9%) were male. A total of 36 patients were enrolled during the initial retrospective phase of the study, while the remaining 9 patients were enrolled in the subsequent prospective phase. Patients with radiologic images affected by significant motion artifacts on chest CT, images in which the neoplasm was too small to be segmented or analyzed, and those with no availability of unenhanced chest CT at baseline were excluded (Figure 2).

The median age at diagnosis was 63 years (18–83 years). As a whole, 42 (93.3%) presented a diagnosis of TC, while 3 (6.7%) presented a diagnosis of AC. The site of primary tumor was the left lung in 19 (42.2%) patients and the right lung in 26 (57.8%), while the primary tumor was centrally and peripherally located in 27 (60%) and 18 (40%), respectively. Only three patients presented a functional syndrome at the diagnosis (carcinoid syndrome in all). According to smoking habits, 28 (62.3%) patients had never been smokers, while 17 (37.7%) were current or former smokers.

Seven patients (15.6%) underwent segmentectomy, thirty-four (75.6%) standard lobectomy, one (2.2%) bilobectomy, and three (6.6%) sleeve lobectomy, one of which Carinal Sleeve Upper Lobectomy.

The postoperative course was uneventful for all the patients. Patients were discharged after a mean of 6 ± 2 days.

According to the AJCC eighth edition staging [29], 33 (73.4%) of the patients had stage I disease at diagnosis, 7 (15.6%) had stage II disease, 4 (8.8%) had stage III disease, and 1 (2.2%) had stage IV disease. Among the pathological characteristics, the median Ki-67 index was 2% (range 1–17), with 34 patients being G1 (75.5%), 11 patients G2 (24.5%), and no one G3; regarding mitotic count, the median count was 1 mitosis/2 mm^2^ (range 1-4); 42 (93.3%) patients had a mitotic index < 2/2 mm^2^, while 3 (6.7%) patients had a mitotic index between 2/2 mm^2^ and 10/2 mm^2^. None had a mitotic count more than 10/2 mm^2^.

At the last follow-up, 41 (91.1%) patients were free of disease with a mean follow-up of 23 months (range 12–87 months). A total of 4 patients (8.9%) experienced disease recurrence. In two patients, the relapse occurred at the mediastinal lymph node level, while in two other patients, it occurred at a distant level and specifically involved the liver and bone in both; half were female, three (75%) had TC, and 1 (25%) had AC. Recurrence occurred at 15, 27, 29, and 40 months, respectively. All the clinicopathological characteristics are summarized in Table 1.

### 3.2. Radiomic Analysis

To assess which factors could be predictors of recurrence, a binary logistic regression analysis of different clinicopathological factors was performed. The following parameters were found to be statistically significant: major age at diagnosis (*p* = 0.020), atypical histotype (*p* = 0.010), advanced stage at diagnosis (*p* = 0.013), lymph node involvement at diagnosis (*p* = 0.006), presence of necrosis (*p* = 0.017), higher Ki-67 (*p* = 0.001), higher tumor grade (*p* = 0.002), higher mitotic count (*p* = 0.006), and presence of functional syndrome (*p* = 0.002). The results are enclosed in Table 2.

**Figure 3:**
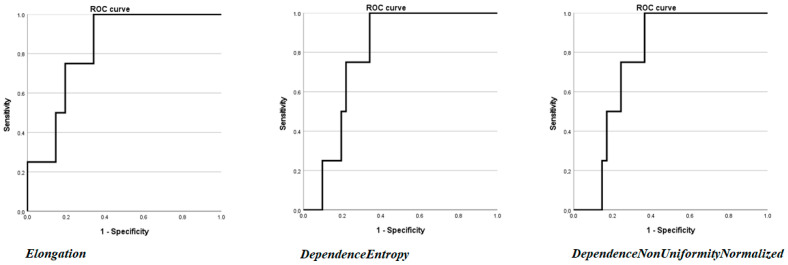
ROC curves of the three radiomics features (DependenceEntropy, DependenceNonUniformityNormalized, and Elongation) found to be statistically significant at univariate binary logistic regression analysis.

Then, univariate analysis was performed by considering the radiomics features. Three features were found to be statistically significant in predicting recurrence. Two features belonged to the GLDM class, namely DependenceEntropy OR (Odds Ratio): 6.649 CI to 95% 1.53–82.35, *p* = 0.049, AUC: 0.784 CI to 95% 0.636–0.892 with a percentage of correctly classified cases of 91.11%; and DependenceNonUniformityNormalized OR: 9.73 × 10^−28^ CI to 95% 2. 89 × 10^−70^–3.27 × 10^−15^, *p* = 0.024, AUC: 0.796 95% CI 0.649–0.901 with a percentage of correctly classified cases of 91.11%. One feature belonged to the 3D shape class, Elongation OR: 0.003 95% CI 0–0.93, *p* = 0.039, AUC: 0.817 95% CI 0.674–0.916 with a percentage of correctly classified cases of 91.11%. The results are presented in Table 3, and the single ROC curve is presented in Figure 3.

## 4. Discussion

The field of radiomics is rapidly flourishing, offering quantitative descriptors that correlate with tumor biology and potentially revolutionize clinical decision-making by providing objective tools to assess tumor aggressiveness non-invasively.

This retrospective/prospective observational study, based on a homogeneous population of patients with lung NETs submitted to radical surgery, proposes different predictive factors of recurrence, both clinicopathological and radiomics features.

Lung neuroendocrine neoplasms (NENs) account for about 25% of all primary pulmonary neoplasms and 30% of all NENs. Mostly represented by neuroendocrine carcinomas, while typical and atypical carcinoids account for 2% and 0.2% of the total, respectively [30]. Despite this, there has been a major increase in incidence [31], with a value of 1.62 new cases per year per 100,000 people according to data from the US SEER registry [2].

The gold standard treatment in localized and locally advanced forms is represented when feasible by radical surgery [7,8]. Generally, the course of lung NETs is indolent; however, at a median follow-up of 54–121 months, a recurrence rate of about 7% in patients with typical carcinoid and up to 35% in those with atypical carcinoid occurs. In one-third of cases, it deals with a local recurrence [9,10]. Disease recurrence often occurs a long time after surgery, and there is still a lack of tools to recognize which clinicopathological or radiological characteristics are most likely predictive of recurrence.

At univariate analysis, using the different clinicopathologic features, the factors that were found to be predictors of recurrence were higher age at diagnosis, an atypical histotype, advanced stage at diagnosis, presence of necrosis, and higher values of proliferation indices (Ki-67 and IM). These data were consistent with previous findings in the literature [5,12,13,15]. These factors, when combined, demarcate the presence of patients with more aggressive disease and greater involvement, who need special attention because they are at greater risk of disease recurrence.

Contrarily, sex was not found to have a statistically significant effect on recurrence; hence, the current findings are contradictory, while it would seem to be a positive predictive factor in some studies [12], this association was not demonstrated in others [32]. Intraparenchymal tumor location, side of the primary tumor, and TTF-1 expression were not found to be prognostic factors.

A study by Guo and colleagues showed that through radiomics, it was possible to differentiate pan-NETs of grade G1/G2 from those of grade G3 [22]. Similarly, Bian and colleagues validated a model for predicting pan-NETs grading from 3T Magnetic Resonance Imaging (MRI) images [33]. In the field of differential diagnosis with pancreatic ductal adenocarcinoma, a model based on CT images showed a predictive value with an AUC of 0.884 [21]. The applications of radiomics have also proven promising in the prognostic of tumor outcome, demonstrating the possibility to discriminate pan-NETs with a short RFS of 36 months from patients with a longer RFS of 84 months [25]. Overall, radiomics is expected to support clinical decision-making rather than replace physicians; it should be viewed as an objective tool to reduce subjective assessment and investigate the deep tumor microenvironment in a preoperative clinical setting. However, radiomics has several limitations, due to variability between scanners, the risk of redundancy, and the lack of validation in a large prospective multicenter study. Therefore, this study can be considered a confirmation of the role of radiomics in neuroendocrine tumors, in line with all other studies described above, but validation with multicenter studies will soon be necessary.

The most significant experiences reported in lung carcinoids have focused mostly on the ability to differentiate these tumors from nodules of other origin, such as hamartomas, using data from CT or PET-18F-FDG images [34,35,36,37]. Cozzi and colleagues retrospectively evaluated images of 27 patients with different pulmonary neuroendocrine neoplasms (TC, AC, neuroendocrine carcinoma), showing statistically significant differences in multiple first-order and higher-order features extracted from contrast medium CT images in correlation with Ki-67. Furthermore, only *Skewness* and *Cluster Shade* were significant for the presence of metastasis [38]. These studies have developed and proposed some promising models, but are limited by small sample sizes.

Our analysis identified three significant radiomic features in predicting disease recurrence (DependenceEntropy, DependenceNonUniformityNormalized, and Elongation). This data suggest that complexity and shape of the tumor are critical determinants of recurrence risk. DependenceEntropy (GLDM) is a measure of the randomness or complexity in the texture, suggesting higher values reflect greater intratumoral heterogeneity, which is often linked to biological aggressiveness. DependenceNonUniformityNormalized (GLDM) measures the variability in the texture pattern. A lower value indicates a more uniform tumor texture. Finally, Elongation (3D Shape) is a shape feature describing how elongated the tumor is. Lower elongation (more spherical tumors) has been linked to differing biological behaviors compared to more irregular shapes.

Analysis of these data showed that the profile of patients affected by lung NET with a higher risk of recurrence is those with an older age at diagnosis, a more advanced stage, and biological characteristics of greater proliferation. At the same time, the use of easily obtainable and available parameters, such as quantitative data achievable from preoperative images, can improve the performance of our risk prediction models.

These results demonstrate how radiomics can be a very promising tool to improve our management and knowledge of neuroendocrine neoplasms. However, it must be emphasized that this observational study has advantages and limitations. Among the advantages is certainly the study design, as a single-center longitudinal (cohort) design allowed us to have less uncertainty with respect to different variables and a better assessment of the impact of individual characteristics. Limitations include the small sample size, the low number of recurrence events, the lack of a validation cohort and consideration of the nature of rare neoplasms, the limits imposed by exclusion criteria, and the short median follow-up period. Despite these limitations, the findings underscore the potential utility of incorporating quantitative radiomics into lung NET management. Identifying high-risk patients pre-surgery could influence surgical extent, guide discussions about adjuvant therapy, and prioritize intensive surveillance schedules post-surgery. To translate these preliminary findings into clinical use, multicenter validation studies with substantially larger patient cohorts are urgently required. These future studies will be able to expand our knowledge by developing predictive models of recurrence using clinical characteristics and radiomic features. They will need to rigorously employ robust methods for feature selection and model training to ensure that the resulting predictive models are stable and generalizable across different scanning protocols and patient populations.

## 5. Conclusions

This study identifies and confirms the predictive markers for individuals with lung NETs undergone radical surgery. As essential clinicopathological criteria to be assessed in the appropriate and individualized treatment of lung NETs, it validates the value of age, histotype, stage, lymph node status, Ki-67 index, mitotic count, and presence of functional syndromes.

Additionally, this work utilizes the innovative tool of radiomics to help identify characteristics that potentially predict recurrence in patients affected by lung NETs. The result is a set of easily obtainable variables that can predict recurrence in a promising way and with accurate performance.

We must, however, emphasize that the small sample size and the low event rate constitute a limitation. Further analyses on larger populations and longer follow-up are required to confirm the observed preliminary data.

## Figures and Tables

**Table 1:** Population characteristics.

Features	*n* = 45 (100%)
*Sex*	
Female	32 (71.1%)
Male	13 (28.9%)
*Median age* (*years*)	63 (18–83)
*Smoking*	
Yes	17 (37.7%)
No	28 (62.3%)
NA	0 (0%)
*Primary tumor localization*	
Peripheral	18 (40.0%)
Central	27 (60.0%)
NA	0 (0%)
*Primary tumor side*	
Left	18 (40.0%)
Right	27 (60.0%)
NA	0 (0%)
*Histotype*	
Typical Carcinoid	42 (93.3%)
Atypical Carcinoid	3 (6.7%)
*Stage at diagnosis*	
I	33 (73.4%)
II	7 (15.6%)
III	4 (8.8%)
IV	1 (2.2%)
NA	0 (0%)
*Nodal status*	
N0	43 (95.5%)
N+	2 (4.5%)
NA	0 (0%)
*Median IM* (/2 mm^2^)	1
*IM* (/2 mm^2^)	
<2	42 (93.3%)
≥2	3 (6.7%)
NA	0 (0%)
*Necrosis*	
Present	3 (6.7%)
Absent	42 (93.3%)
NA	0 (0%)
*Median Ki67* (%)	2
*Grading for Ki67* (%)	
1–2	34 (75.5%)
3–19	11 (24.5%)
>20	0 (0%)
NA	0 (0%)
*Recurrence*	
Yes	4 (8.9%)
No	41 (91.1%)
*Alive*	
Yes	45 (100%)
No	0 (0%)

NA: not available.

**Table 2:** Univariate logistic regression analysis for the clinicopathological features.

Features	OR	CI 95%	*p*-Value
Major age at diagnosis	1.115	1.017–1.222	0.020
Atypical histotype	7.867	1.653–37.441	0.010
Presence of functional syndrome	20.667	3.113–137.206	0.002
Major stage at diagnosis	2.928	1.254–6.833	0.013
Presence of necrosis	8.714	1.468–51.737	0.017
Higher Ki-67	2.274	1.393–3.712	0.001
Higher grading	27.726	3.284–234.099	0.002
Higher mitotic count	14.000	2.105–93.109	0.006
Pathologic lymph node	14.250	2.143–94.741	0.006

OR: Odds ratio.

**Table 3:** Univariate logistic regression analysis for the radiomics features (SD: Standard Deviation).

Features	Recurrence	Non-Recurrence	OR (CI 95%)	AUC (CI 95%)	Correctly Classified Cases	*p*-Value
Non-contrast Phase	Average + SD	Average + SD				
DependenceEntropy (GLDM)	5.3026 + 0.1856	4.516 + 0.7748	6.649 (1.53–82.35)	0.784 (0.636–0892)	91.11%	0.049
DependenceNonUniformityNormalized (GLDM)	0.0490 + 0.0085	0.1248 + 0.1227	9.73 × 10^−28^ (2.89 10^−70^–3.27 × 10^−15^)	0.796 (0.649–0.901)	91.11%	0.024
Elongation (3D Shape)	0.6380 + 0.1954	0.7949 + 0.1357	0.003 (0–0.93)	0.817 (0.674–0.916)	91.11%	0.039

## Data Availability

All relevant data are within the manuscript.
